# The Voluntary Medical Institutions of Bristol

**Published:** 1984-07

**Authors:** 


### THE VOLUNTARY MEDICAL INSTITUTIONS OF BRISTOL

**C. Bruce Perry**

**Price 90 p.***

This pamphlet is no 52 of a series produced by the Bristol Branch of the Historical Association, which deal with all aspects of Bristol's history. It has 22 pages and contains some beautiful illustrations. It deals with the history of those Bristol Medical Institutions which had a voluntary origin, The Royal Infirmary, the General Hospital, the Maternity Hospital, The Royal Hospital for Sick Children etc as well as some of the institutions which have ceased to exist such as the Dispensaries. It includes Bristol's most recent Voluntary Medical Institution, St Peter's Hospice. No mention is made of course of those institutions which are of municipal or state origin such as Southmead or Frenchay Hospitals. The booklet begins 'In the early 18th century the problem of the sick poor was impinging on the public conscience' and the underlying theme seems to be the response to this problem. In his final paragraph the author speculates on the motives of the men who were the founders of these institutions. He seems to conclude that if self interest played a part it was usually enlightened. It seems churlish to complain that this little book would be more useful for reference purposes if provided with an index and paragraph headings. We must be grateful to Professor Perry for once again giving perspective to the Bristol Medical scene.

M.G.W.

*Obtainable from the Department of History, Bristol University or most Bristol bookshops.

